# Patterns of Digital Media Engagement During Wartime and Their Associations With Stress, Resilience, and Well-Being: Cross-Sectional Survey Study

**DOI:** 10.2196/91484

**Published:** 2026-06-17

**Authors:** Moti Zwilling, Galit Klein, Sharon Barak, Noa Shtainmetz, Riki Tesler, Avi Zigdon

**Affiliations:** 1 Department of Economics and Business Administration Ariel University Ariel, Judea and Samaria Area Israel; 2 Ariel cyber innovation center Ariel Israel; 3 Faculty of Health Sciences Department of Nursing Ariel University Ariel Israel; 4 Faculty of Health Sciences Department of Health Systems Management Ariel University Ariel, Judea and Samaria Area Israel

**Keywords:** digital media engagement, institutional engagement, resilience, stress, wartime, well-being

## Abstract

**Background:**

During wartime, civilians rely on digital media for information, connection, and coping. However, engagement patterns may differ in their associations with stress, resilience, and well-being.

**Objective:**

This study aimed to characterize patterns of digital media engagement during wartime and examine their associations with stress, resilience, and well-being in a cross-sectional survey of Israeli adults.

**Methods:**

We conducted a cross-sectional web-based survey in Israel in early January 2024. Participants (N=512; age: range18-65, mean 37.54, SD 12.89 years; 241/512, 47.1% male) were recruited using nonprobability quota sampling. Well-being was measured using the 5-item World Health Organization Well-Being Index, stress using the 21-item Depression Anxiety Stress Scales stress subscale, and resilience using the 10-item Connor-Davidson Resilience Scale. Participants reported the extent to which their use of 10 digital media activities increased since the beginning of the war (5-point Likert scale). Analyses included 2-step cluster analysis, exploratory factor analysis (principal axis factoring with Promax rotation), and structural equation modeling path analysis.

**Results:**

Cluster analysis identified 2 engagement profiles: low engagement (126/512, 24.6%) and high engagement (386/512, 75.4%; silhouette coefficient=0.30). The high-engagement profile reported higher stress (median 2.39, 95% CI 2.31-2.47) than the low-engagement profile (median 2.01, 95% CI 1.87-2.15; *P*<.001). Exploratory factor analysis supported a 3-factor structure (active, passive, and institutional engagement), explaining 61.86% of the variance (Kaiser-Meyer-Olkin=0.77; Bartlett *χ*^2^_36_=1007.6; *P*<.001). Structural equation modeling showed acceptable fit (*χ*^2^_1_=1.5; *P*=.21; Comparative Fit Index=0.999; Tucker-Lewis Index=0.981; standardized root-mean-square residual=0.013; root-mean-square error of approximation=0.033). Resilience was negatively associated with stress (*β*=−0.24, 95% CI −0.34 to −0.14; *P*<.001) and positively associated with well-being (*β*=0.30, 95% CI 0.18-0.42; *P*<.001). Stress was positively associated with active (*β*=0.27, 95% CI 0.17-0.37; *P*<.001), passive (*β*=0.31, 95% CI 0.21-0.41; *P*<.001), and institutional engagement (*β*=0.16, 95% CI 0.06-0.26; *P*<.001). Active engagement was positively associated with well-being (*β*=0.12, 95% CI 0.04-0.20; *P*=.006), passive engagement showed a negative trend (*β*=−0.08, 95% CI −0.18 to 0.02; *P*=.08), and institutional engagement was not significantly associated with well-being (*β*=0.07, 95% CI −0.01 to 0.15; *P*=.11). The total indirect association between resilience and well-being through stress and engagement was significant (indirect effect=0.09, 95% bootstrap CI 0.05-0.13).

**Conclusions:**

Wartime digital media engagement clustered into distinct profiles and dimensions, each showing different associations with stress, resilience, and well-being. This study advances the field by empirically distinguishing active, passive, and institutional engagement, with the latter often overlooked in crisis-media research. The findings offer a more detailed framework for understanding how civilians navigate digital environments during an ongoing armed conflict and provide practical implications for psychosocial guidance and crisis communication, encouraging more intentional engagement and reducing excessive passive consumption. Causal inferences are limited by the cross-sectional design.

## Introduction

### Overview

The digital landscape has become an integral component of modern conflict, significantly shaping how individuals and nations navigate and respond to crises [1]. During wartime, civilians increasingly rely on digital channels, including news websites, social media, messaging applications, and institutional platforms, for information, social connection, and emotional regulation; this engagement often involves exposure to emotionally saturated content with implications for digital well-being [2-4]. Real-time social media updates have increasingly displaced traditional broadcast media as the main vehicle for collective exposure to traumatic events [5,6]. The October 7, 2023, attack on Israel exemplified this shift, when platforms such as Telegram and WhatsApp became dominant channels through which civilians encountered graphic wartime content, often involuntarily [5,7,8].

Wartime digital media engagement is heterogeneous, ranging from passive consumption (eg, continuous news monitoring and “doomscrolling”) to active engagement involving information dissemination and seeking social support, and may shape coping resources and mental state [9-13]. Evidence from collective crises indicates that trauma-related media exposure is associated with acute stress and functional impairment [14], with self-reinforcing cycles in which greater concerns lead to more media coverage and higher distress [15-17]. During COVID-19, higher exposure was linked to higher depressive (Patient Health Questionnaire-9 [PHQ-9]) and anxiety (Generalized Anxiety Disorder-7 Scale [GAD-7]) symptom scores [18], and during the Russia–Ukraine war, perceived negative media impact was linked to increased anxiety and reduced resilience, even among uninvolved populations [19,20]. The active vs passive distinction is central to digital well-being research [21-23], although operationalizations vary widely and associations depend on measurement and contextual factors, including platform type [22-25].

Israel has a long-standing research tradition on media-based exposure to terrorism and related mental health consequences [26]. Following the October 7, 2023, attack, a nationwide prospective study documented marked increases in posttraumatic stress disorder (PTSD), generalized anxiety, and depression among Israeli adults [27], and extensive media consumption, particularly graphic Telegram content, was identified as a significant risk factor for PTSD among indirectly exposed Israelis [8]. Psychological stress has been consistently linked to increased media engagement during crises: anxiety related to health-protective behaviors and information seeking is associated with greater media exposure during emergencies [15,16,28], uncertainty-inducing news representations may sustain cycles of worry and excessive consumption [29], and acute distress may override cognitive resources in shaping digital behavior [16,30].

Resilience, conceptualized as the maintenance or rapid recovery of mental health after stressor exposure, may buffer the psychological effects of crisis-related media exposure [31] and moderate associations between crisis-related information exposure and emotional distress [32], including effects of personality vulnerabilities during the COVID-19 pandemic [33]. In Israel, resilience-related factors such as emotion regulation and perceived social support showed stronger associations with mental health outcomes than social media use itself [34].

Protection motivation theory (PMT) provides a conceptual framework, proposing that protective responses depend on appraisals of threat severity and vulnerability and on coping appraisals such as response efficacy and self-efficacy [35,36]. PMT has been applied in crisis contexts; a meta-analysis found positive associations between PMT components and protective behaviors during COVID-19, with coping appraisal variables emerging as particularly strong correlates [36]. Extending PMT to media-related processes, trust in media channels has been shown to predict both threat and coping appraisals [37], and perceived risk and eHealth literacy have been linked to engagement with social media health content and protective behavior [38].

Despite growing evidence, important gaps remain. Most studies focus on overall exposure without differentiating engagement patterns such as active, passive, and institutional modes [22,23]; fewer examine engagement during active armed conflict [19,20]; and resilience has been examined as a moderator in only a limited number of studies, with few examining all 3 engagement patterns simultaneously [32,33]. This study addresses these gaps by empirically characterizing distinct patterns of wartime digital media engagement (active, passive, and institutional) and examining their differential associations with stress, resilience, and well-being. By incorporating institutional engagement, it provides a more granular account of how civilians navigate digital environments during ongoing armed conflict. Based on the framework outlined above, this study addressed the following research questions (RQs):

- RQ1: are there distinct profiles of digital media engagement during wartime, and do these profiles differ in stress levels and well-being?
- RQ2: what is the underlying factor structure of digital media engagement behaviors during a national crisis?
- RQ3: how are stress and resilience associated with well-being during wartime, and do engagement types (active, passive, and institutional) relate to this association?

### Objectives

This study aimed to characterize patterns of digital media engagement during wartime and examine their associations with stress, resilience, and well-being in a cross-sectional survey. We also aimed to identify distinct engagement patterns (including active, passive, and institutional modes) and assess how these patterns relate to psychological outcomes in the context of an acute armed conflict.

## Methods

### Study Design and Participants

We conducted a cross-sectional web-based survey to examine patterns of digital media engagement and their associations with stress, resilience, and well-being during a national crisis. Data collection took place in early January 2024, during the fourth month of the ongoing war, using research interfaces built on the Qualtrics platform. Participants were recruited through Panel4all, a professional web-based survey firm, using an online panel. The survey firm distributed invitations via a unique survey link sent to panel members. Recruitment followed a nonprobability quota sampling approach designed to approximate the distribution of Israeli adults aged 18 to 65 years by age, gender, level of religiosity, and district of residence, as specified by the survey firm. Panel members received incentives for survey completion, provided by the survey firm as part of its standard rewards program (eg, points redeemable for online shopping vouchers).

### Procedure

The survey introduction described the study objectives, provided the researcher’s contact information, assured anonymity, and clarified that responses would be used for research purposes. Participants provided electronic informed consent before accessing the questionnaire. Participation was voluntary, and participants could withdraw at any time without penalty or consequences.

### Sample

The final sample consisted of 512 adults aged 18 to 65 years (mean age 37.54, SD 12.89 years), with 47.1% (241/512) identifying as male. A total of 64% (330/512) were employees, 8.4% (43/512) were managers, 4.5% (23/512) were furloughed, and 9% (46/512) were unemployed. Regarding the sector, 51.2% (262/512) were employed in private companies and 24.6% (126/512) in public organizations; the remaining participants worked in the military or police force. A total of 18.8% (96/512) reported cyber-related knowledge, either through a computer science degree or employment in a related field. Table 1 summarizes participants’ demographic and background characteristics.

**Table 1:** Demographic and background characteristics of the participants (N=512).

Variable		Value
**Sex, n (%)**		
	Male	241 (47.1)
	Female	271 (52.9)
Age (years), mean (SD); range		37.54 (12.89); 18-65
**Employment status, n (%)**		
	Employee	330 (64.5)
	Manager	43 (8.4)
	Furloughed	23 (4.5)
	Unemployed	46 (9)
	Other	70 (13.7)
**Cyber knowledge^a^, n (%)**		
	Yes	96 (18.8)
	No	416 (81.3)
**Occupation, n (%)**		
	Public organization	126 (24.6)
	Private company	262 (51.2)
	Military or security organization	30 (5.9)
	Unemployed or other	94 (18.4)

^a^Cyber knowledge—Did you learn or work in the cyber area (eg, software engineering, computer science, information technology, quality assurance, and software technician)?

The data were collected by an external survey company that does not provide datasets with missing responses; accordingly, the dataset used in this study did not contain missing values.

### Response Rate and Data Cleaning

**Figure 1:**
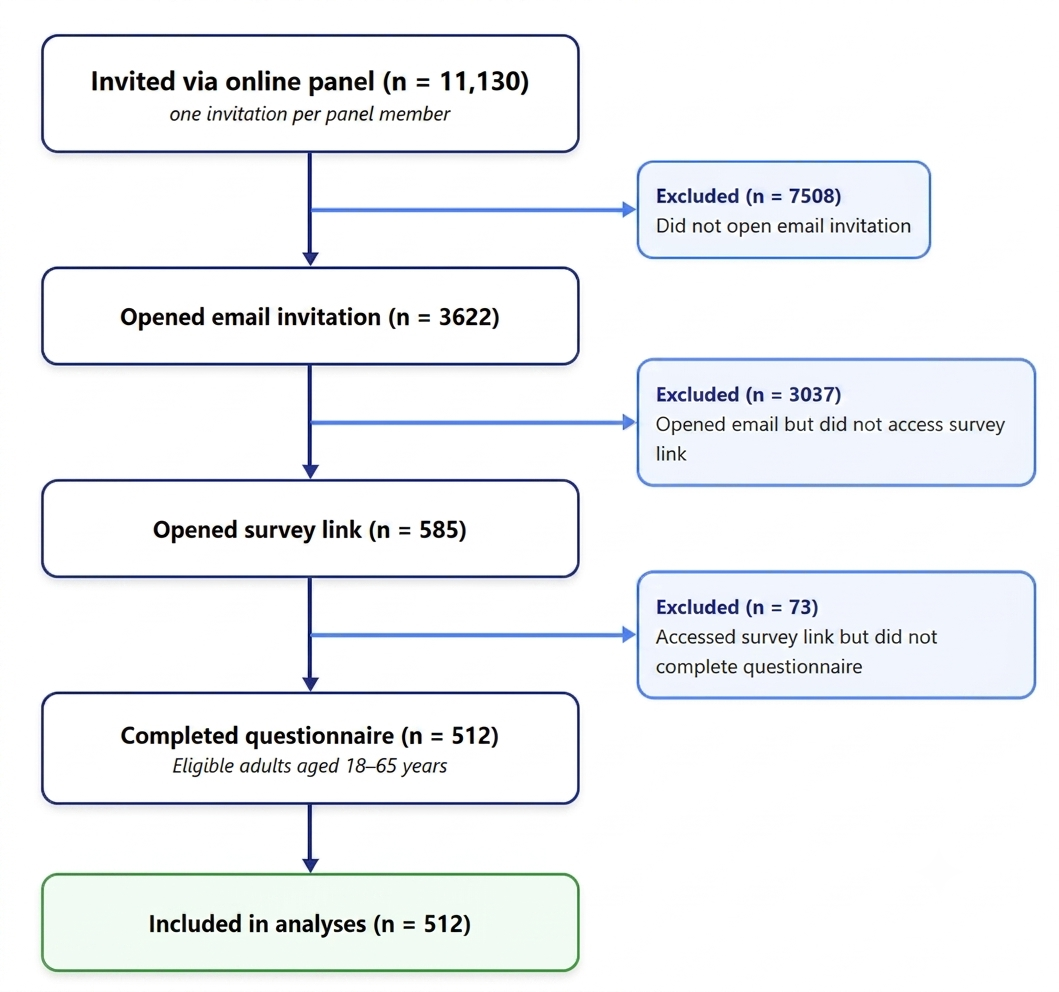
Flowchart of participant selection for analysis.

A total of 11,130 email invitations were sent (1 invitation per panel member); of which 3622 invitations were opened, 585 respondents accessed the survey link, and 512 completed the questionnaire and were included in the analyses. Respondents who accessed the link but did not complete the questionnaire (n=73) were not included (Figure 1).

### Measures

#### Well-Being

Well-being was measured using the World Health Organization Well-Being Index, which includes 5 items (eg, “I have felt cheerful and in good spirits”; Cronbach α=0.87) rated on a 6-point Likert scale (0=“not at all” to 5=“all the time”) [39].

#### Stress

Stress was measured using the 21-item Depression Anxiety Stress Scales 21 [40,41]. We used only the 7-item stress subscale (eg, “I tended to over-react to situations”; Cronbach α=0.92) rated on a 4-point Likert scale (0=“did not apply to me at all” to 3=“applied to me very much, or most of the time”) [42].

#### Resilience

Resilience was measured using the short version of the Connor-Davidson Resilience Scale (Cronbach α=0.88) [43].

#### Media Usage Assessment

A distinction between active and passive media use has been proposed as a way to help explain why some individuals may experience better or worse psychological outcomes from social media usage [44]. Although there has been considerable discussion regarding how to best operationalize and measure active and passive social media usage [45], studies typically ask participants to self-report how often they engage in a variety of active and passive activities. Active use is generally defined as interacting with other users (eg, creating posts or status updates) and directed communication (eg, sending private messages), whereas passive use refers to consuming media content without interacting with others, such as scrolling through newsfeeds [44]. In this study, media usage was assessed across 10 categories designed to capture both active and passive digital behaviors in the context of a national crisis. Participants were asked to describe the extent to which they experienced an increase in their usage of various media types since the beginning of the war (eg, “browsing news websites on a phone or computer”) [46]. Responses were rated on a 5-point Likert scale (1=“not at all” to 5=“to a very great extent”).

Table 2 summarizes the participants’ media behaviors and psychological characteristics during the initial period of the war.

**Table 2:** Participants’ media behaviors and psychological characteristics. Mean media usage frequencies and psychological characteristics among Israeli adults (N=512) surveyed during the Israel–Hamas war (January 2024). Media usage was rated on a 5-point Likert scale (1=“not at all” to 5=“to a very great extent”). Psychological characteristics are presented as medians with 95% CIs.

Variable		Value
**Media usage, mean (95% CI)**		
	Browsing news websites on a phone or computer	4.04 (3.9-4.1)
	Browsing social media	3.83 (3.7-3.9)
	Sharing posts on social media	2.79 (2.6-2.9)
	Joining new groups on WhatsApp	2.62 (2.5-2.7)
	Signing digital petitions	2.41 (2.3-2.5)
	Transferring money for donations through digital platforms	2.41 (2.2-2.4)
	Accessing the digital banking website	2.30 (3.1-3.4)
	Accessing government and public authority websites	3.27 (2.7-3.00)
	Listening to podcasts	2.86 (2.2-2.5)
	Watching videos	2.34 (3.4-3.6)
	Total media usage	3.00 (2.9-3.06)
**Psychological characteristics, median (95% CI)**		
	Stress	2.3 (2.2-2.4)
	Resilience	3.6 (3.5-3.7)
	Well-being	3.0 (2.9-3.1)

#### Demographic Questionnaire

Participants provided information about their age, gender, employment status, type of organization they worked for, and their cyber-related knowledge. Cyber knowledge was defined as either holding a degree in computer science or working in a computer science–related field (eg, software engineering, computer science, IT, quality assurance, and software technician) [47].

### Statistical Analysis

Statistical analyses were performed using a multistage analytic strategy. First, descriptive statistics were calculated for all media usage and psychological variables, including means, medians, and 95% CIs. To identify patterns of media engagement among participants, a 2-step cluster analysis was conducted. We selected a 2-step cluster analysis because it is appropriate for large datasets and enables automatic determination of the optimal number of clusters. The adequacy of the 2-step cluster analysis was also assessed using the silhouette value. Group differences between the high- and low-media engagement clusters were examined using independent samples 2-tailed *t* tests for continuous variables and chi-square tests for categorical variables. Effect sizes for between-group differences were calculated using Cohen *d*, defined as the difference between group means divided by the pooled SD. Effect sizes were interpreted using conventional benchmarks (0.2=small, 0.5=medium, and 0.8=large) [48]. In the clustering analysis, media engagement was treated as a categorical variable (low vs high), as this approach provides clinically and practically meaningful profiles and allows clearer interpretation of behavioral patterns during crisis conditions. To explore the underlying structure of media usage, an exploratory factor analysis (EFA) using principal axis factoring with Promax rotation was performed. Factor retention was based on eigenvalues greater than 1, and sampling adequacy was confirmed via the Kaiser-Meyer-Olkin (KMO) measure and Bartlett test of sphericity. Reliability analyses guided the exclusion of items with low loadings. Subsequently, hierarchical stepwise regression analyses were conducted to assess the contribution of demographic variables, types of media usage, stress, and resilience to individual well-being. Finally, to examine the hypothesized mediation and moderation effects, path analysis using structural equation modeling (SEM) was performed. Model fit was evaluated using standard indices, including chi-square, comparative fit index (CFI), Tucker-Lewis Index (TLI), standardized root-mean-square residual (SRMR), and root-mean-square error of approximation (RMSEA). Indirect effects were estimated with bias-corrected CIs to assess the significance of mediated pathways.

### Ethical Considerations

The study protocol received full ethical approval from the Ethics Committee of Ariel University (institutional review board approval number AU-SOC-MZ-20231203). Before participation, respondents viewed an online information sheet describing the study aims and procedures, expected duration, potential risks and benefits, and the voluntary nature of participation. Participants then provided electronic informed consent before accessing the questionnaire. The survey was administered anonymously, and no directly identifying information was collected. The anonymized data were stored in a restricted-access environment and were accessible only to the research team. Participants received panel-based incentives for survey completion through the survey firm’s standard rewards program. The research team did not provide any direct financial compensation. No images or materials containing identifiable participant information are included in this manuscript or its supplementary files.

## Results

**Figure 2:**
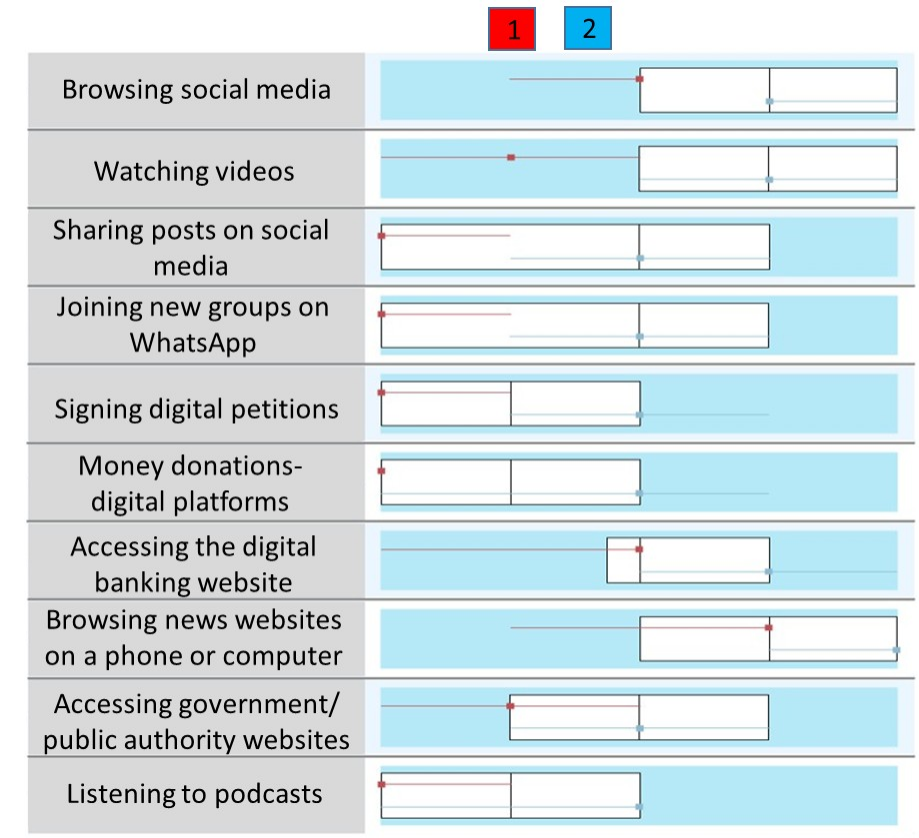
Box-and-whisker plots of the distribution of media engagement variables across the identified clusters.

We conducted a 2-step cluster analysis. The 2-cluster solution demonstrated an acceptable silhouette coefficient (0.30), indicating fair separation between clusters. Alternative cluster solutions, such as a 3-cluster solution, were also examined. While additional clusters could be extracted, these solutions resulted in reduced interpretability and overlapping profiles without meaningful improvement in cluster quality indices. Therefore, the 2-cluster solution was retained as the most parsimonious and theoretically meaningful representation of the data. The first cluster exhibited significantly higher media usage and was classified as the “high-media engagement” group, while the second cluster was identified as the “low-media engagement” group, as illustrated in Figure 2.

Figure 2 depicts Box-and-whisker plots displaying the distribution of 10 digital media usage behaviors across 2 engagement clusters identified via a 2-step cluster analysis among Israeli adults (N=512) surveyed during the Israel–Hamas war (January 2024). Red boxes represent the high-media engagement cluster (n=386) and blue boxes represent the low-media engagement cluster (n=126). Media usage was rated on a 5-point Likert scale (1=“not at all” to 5=“to a very great extent”).

An independent samples *t* test and chi-square test were conducted to examine between-cluster differences in media usage behaviors (Table 3), as well as differences in demographic and psychological characteristics (Table 4).

**Table 3:** Between-cluster differences in media usage behaviors among Israeli adults (N=512) surveyed during the Israel–Hamas war (January 2024). A 2-step cluster analysis identified 2 engagement profiles: low-media engagement (cluster 1) and high-media engagement (cluster 2). Group differences were examined using independent samples 2-tailed t tests. Effect sizes for between-group differences were calculated using Cohen d, defined as the difference between group means divided by the pooled SD. Effect sizes were interpreted using conventional benchmarks (0.2=small, 0.5=medium, and 0.8=large) [48].

Variables composing the clusters	Cluster 1: low-media engagement (n=126), mean (95% CI)	Cluster 2: high-media engagement (n=386), mean (95% CI)	Between-group differences		
			*t* test (*df*)	Cohen *d*	*P* value
Browsing news websites	3.28 (3.02-3.54)	4.29 (4.20-4.38)	–9.12 (510)	–0.80	<.001
Browsing social media websites	2.72 (2.51-2.93)	4.19 (4.10-4.28)	–14.56 (510)	–1.28	<.001
Sharing posts on social media	1.52 (1.36- 1.67)	3.20 (3.06-3.34)	–12.64 (510)	–1.11	<.001
Joining new WhatsApp groups	1.57 (1.41-1.73)	2.96 (2.83-3.09)	–11.17 (510)	–0.98	<.001
Signing digital petitions	1.40 (1.26-1.53)	2.74 (2.61-2.88)	–10.66 (510)	–0.94	<.001
Transferring funds for donations via digital means	1.37 (1.23-1.50)	2.61 (2.47-2.74)	–9.80 (510)	–0.86	<.001
Browsing the digital banking websites	2.37 (2.14-2.59)	3.56 (3.44- 3.69)	–9.48 (510)	–0.83	<.001
Browsing government and public authority websites	2.17 (1.95-2.40)	3.09 (2.95-3.22)	–6.78 (510)	–0.60	<.001
Listening to podcasts	1.69 (1.49-1.88)	2.55 (2.41-2.69)	–6.41 (510)	–0.56	<.001
Watching videos online	2.29 (2.09-2.2.50)	3.96 (3.83-4.05)	–14.32 (510)	–1.26	<.001

**Table 4:** Between-cluster differences in demographic and psychological characteristics among Israeli adults (N=512) surveyed during the Israel–Hamas war (January 2024). A 2-step cluster analysis identified 2 engagement profiles: low-media engagement (cluster 1) and high-media engagement (cluster 2). Group differences were examined using independent samples 2-tailed t tests for continuous variables and chi-square tests for categorical variables.

Characteristic				Cluster 1: low-media engagement (n=126)		Cluster 2: high-media engagement (n=386)		Between-group differences		
								*t* test (*df*) or chi-square (*df*)		*P* value
**Age (years)**										
	Mean (95% CI)			31.12 (36.81- 41.43)		37.02 (35.74-38.30)		1.59 (510)^a^		.11
**Sex, n (%)**								14.8 (1)^b^		<.001
	Female			48.0 (38.1)		223.0 (57.8)				
	Male			78.0 (61.9)		163.0 (42.2)				
**Work-related characteristics, n (%)**										
	**Occupation**							0.7 (4)^b^		.67
		Employee	72 (62.7)		251 (65)					
		Manager	12 (9.5)		31 (8)					
		Furloughed	5 (4.0)		18 (4.7)					
		Unemployed	11 (8.7)		35 (9.1)					
		Other	19 (15.1)		51 (13.2)					
	**Company type**							0.2 (3)^b^		.24
		Private company	63 (50)		199 (51.6)					
		Public sector	25 (19.8)		101 (26.2)					
		Military or security	10 (7.9)		20 (5.2)					
		Unemployed	28 (22.2)		66 (17.1)					
**Cyber knowledge**								0.2 (1)^b^		.67
	Yes			22 (17.5)		74 (19.2)				
	No			104 (82.5)		312 (80.8)				
**Psychological characteristics, median (95% CI)**										
	Stress			2.01 (1.87-2.15)		2.39 (2.31-2.47)		–4.59 (510)^a^		<.001
	Resilience			3.58 (3.5-3.7)		3.58 (3.5-3.6)		0.03 (510)^a^		.98
	Well-being			3.1 (2.8-3.2)		3.0 (2.9-3.1)		0.43 (510)^a^		.67

^a^Indicates a *t* test statistic.

^b^Indicates a Chi-square statistic.

The results confirmed a significant difference between participants with low-media engagement (cluster 1) during the initial stages of the war and those with high-media engagement (cluster 2) across all media usage categories. Participants in the high-media engagement cluster spent more time browsing for information and news, sending WhatsApp messages, watching videos, and listening to podcasts. They also displayed more active behaviors, such as signing petitions, making donations through websites, sharing posts on social media, and accessing government and banking sites more frequently. Overall, between-cluster differences in media usage behaviors ranged from 0.56 (listening to podcasts) to 1.28 (browsing social media), indicating moderate to large effect sizes (Table 3).

In addition to media usage, the only other characteristic that significantly differed between the 2 clusters was the level of stress. Participants in the high-media engagement cluster reported higher stress levels, indicating that higher stress was associated with greater media engagement (Table 4). The between-cluster differences in media usage and psychological variables further support the validity of the 2-cluster solution.

Our next analysis explored the connection between stress, media usage, and well-being. To examine the structure of media usage, we first conducted an EFA using principal axis factoring with oblique (Promax) rotation. Factors were identified based on eigenvalues greater than 1 [49,50]. The EFA revealed a 3-factor structure among the 10 items (Table 5). Based on previous studies, we expected a 2-cluster structure; however, the EFA indicated a 3-cluster solution. The 3 factors accounted for 61.86% of the total variance, which is considered adequate in social science research [51]. The KMO measure of sampling adequacy was 0.77, and Bartlett test of sphericity was significant (*χ*^2^_36_=1007.6; *P*<.001), confirming the suitability of the data for factor analysis [52]. Factor loadings ranged from 0.57 to 0.82, indicating a strong contribution of the items to their respective factors. Ultimately, the 3 factors were identified as active media usage, passive media usage, and institutional media usage (Table 5).

**Table 5:** Factor analysis for the structure of media usage. An exploratory factor analysis of 10 digital media usage behaviors among Israeli adults (N=512) surveyed during the Israel–Hamas war (January 2024) was conducted using principal axis factoring with Promax rotation. Three factors were identified: active usage, passive usage, and institutional usage. Total variance explained=61.86%; KMO=0.77; Bartlett test χ236=1007.6; *P*<.001. Factor loadings below 0.40 are suppressed. “Listening to podcasts” was excluded based on low reliability.

Items	Factor 1: active usage	Factor 2: passive usage	Factor 3: institutional usage
Signing digital petitions	0.80^a^	0.18^b^	0.05^b^
Sharing posts on social media	0.75^a^	0.22^b^	0.08^b^
Joining new groups on WhatsApp	0.69^a^	0.19^b^	0.06^b^
Transferring money for donations through digital platforms	0.64^a^	0.15^b^	0.12^b^
Browsing news websites on a phone of computer	0.21^b^	0.88^a^	0.17^b^
Browsing social media	0.25^b^	0.78^a^	0.09^b^
Watching videos	0.18^b^	0.61^a^	0.14^b^
Accessing government and public authority websites	0.06^b^	0.20^b^	0.87^a^
Accessing the digital banking websites	0.09^b^	0.17^b^	0.77^a^
Cronbach α	0.71^b^	0.68^b^	0.63^b^

^a^Values indicate primary factor loadings and define item membership in each factor.

^b^Values represent cross-loadings and were not used to assign items to factors.

Next, we examined the contribution of different types of media use to individuals’ well-being. Tables 6 and 7 present the results of a 3-step hierarchical regression analysis. In step 1, demographic variables were entered as control variables. In step 2, the different categories of media use were added to the model. In step 3, stress and resilience were included to assess their incremental contribution to well-being.

**Table 6:** Hierarchical regression results: well-being as the dependent variable. Hierarchical multiple regression analysis predicting psychological well-being (5-item World Health Organization Well-Being Index) among Israeli adults (N=512) surveyed during the Israel–Hamas war (January 2024). Model 1.1 includes demographic variables, Model 1.2 adds 3 types of media usage, and Model 1.3 adds stress and resilience. The *P* values are 2-tailed.

Variables		Model 1.1			Model 1.2			Model 1.3	
		*β* (95% CI)	*P* value	*β* (95% CI)		*P* value	*β* (95% CI)		*P* value
**Step 1: demographic variables**									
	Sex (1-male, 2-female)	–0.17 (–0.52 to –0.18)	.001	–0.17 (–0.51 to –0.16)		>.001	–0.08 (–0.31 to 0.01)		.04
	Age	–0.19 (–0.02 to –0.01)	>.001	–0.19 (–0.02 to –0.01)		>.001	–0.25 (–0.02 to –0.01)		>.001
**Step 2: media usage**									
	Active usage	—^a^	—	0.05 (–0.04 to 0.14)		.31	0.12 (0.03 to 0.20)		<.001
	Passive usage	—	—	–0.09 (–0.19 to 0.01)		.07	–0.07 (–0.16 to 0.02		.08
	Institutional usage	—	—	0.10 (0.00 to 0.19)		.04	0.06 (–0.00 to 0.14)		.11
**Step 3: psychological characteristics**									
	Stress	—	—	—		—	–0.29 (–0.46 to –0.25)		<.001
	Resilience	—	—	—		—	0.32 (0.34 to 0.56)		<.001

^a^Not applicable.

**Table 7:** Hierarchical regression model fit statistics for psychological well-being.

Statistic	Model 1.1	Model 1.2	Model 1.3
*R* ^2^	0.064	0.07	0.26
Δ*R*^2^	—^a^	0.02^b^	0.20^b^
*F* test (*df*)	18.42 (2, 509)^b^	8.82 (5, 506)^b^	28.78 (7, 504)^b^

^a^Not applicable/Not available.

^b^Indicates *P*<.001.

Model 1.1 indicates that females and younger individuals reported higher well-being compared with males and older participants (*β*=−0.17, 95% CI −0.52 to −0.18; *β*=−0.19, 95% CI −0.02 to −0.01, respectively; both *P*<.001). Adding media usage variables in Model 1.2 revealed that institutional media usage was positively associated with well-being (*β*=0.10, 95% CI 0.00 to 0.19; *P*=.04), whereas passive media usage showed a marginally negative association (*β*=−0.09, 95% CI −0.19 to 0.01; *P*=.07). Active media usage was not significantly associated with well-being at this stage (*β*=0.05, 95% CI −0.04 to 0.14; *P*=.31). Including psychological variables substantially improved the model, increasing the explained variance from 7% to 26% (Δ*R*^2^=0.20, *F*_7,504_=28.78; *P*<.001). Both stress and resilience significantly predicted well-being (*β*=−0.29, 95% CI −0.46 to −0.25; *β*=0.32, 95% CI 0.34-0.56, respectively; both *P*<.001). In addition, active media usage became a significant positive predictor of well-being (*β*=0.12, 95% CI 0.03-0.20; *P*<.001). Overall, most statistically significant effects were accompanied by CIs that did not include zero, indicating reliable associations. In contrast, the CIs for passive media usage crossed zero, and the interval for institutional media usage approached zero, suggesting greater uncertainty regarding these relationships.

Lastly, our main hypothesis proposed that media usage moderates the relationship between resilience, stress, and well-being. To test this hypothesis, we conducted path analyses. The model specified the relationships among resilience, stress, active media usage, passive media usage, institutional media usage, and participants’ well-being. Detailed results of the path analyses are presented in Table 8.

**Table 8:** Path analysis results examined the relationships among resilience, stress, media usage (active, passive, and institutional), and well-being among Israeli adults (N=512) surveyed during the Israel–Hamas war (January 2024). The model was estimated using structural equation modeling. Unstandardized coefficients (B) are reported with SE and 95% CIs. Indirect effects were estimated using bias-corrected bootstrap CIs. Model fit: χ21=1.5; *P*=.21; comparative fit index=0.999; Tucker-Lewis Index=0.981; standardized root-mean-square residual=0.013; root-mean-square error of approximation=0.033. The *P* values are 2-tailed.

Path		B (95% CI)	SE	*P* Value
**Direct effects**				
	Resilience → stress	–0.28 (–0.38 to –0.18)	0.05	<.001
	Stress → active media usage	0.33 (0.23 to 0.43)	0.05	<.001
	Stress→ passive media usage	0.37 (0.27 to 0.47)	0.05	<.001
	Stress → institutional media usage	0.20 (0.10 to 0.30)	0.05	<.001
	Active media usage → well-being	0.12 (0.04 to 0.20)	0.04	<.001
	Passive media usage → well-being	–0.08 (–0.18 to 0.02)	0.05	.08
	Institutional media usage → well-being	0.07 (–0.01 to 0.15)	0.04	.11
	Resilience → well-being	0.43 (0.31 to 0.55)	0.05	<.001
	Stress → well-being	–0.33 (–0.43 to –0.23)	0.05	<.001
**Indirect effects (bootstrap)**				
	Resilience → stress→ media usage→ well-being	0.09 (0.05 to 0.13)	—^a^	—
	Resilience → stress→ active media usage→ well-being	–0.01 (–0.02 to –0.01)	—	—
	Resilience → stress→ passive media usage→ well-being	0.01 (–0.01 to 0.09)	—	—
	Resilience → stress→ institutional media usage→ well-being	–0.01 (–0.02 to –0.01)	—	—

^a^Not applicable.

The findings revealed an acceptable fit of the data (*χ*^2^_1_=1.54; *P*=.21; CFI=0.999; TLI=0.981; SRMR=0.013; RMSEA=0.033). Resilience was negatively associated with stress (*β*=–0.24, SE=0.05; *P*<.001) and positively associated with well-being (*β*=0.30; SE=0.06; *P*<.001). Stress was positively associated with active media usage (*β*=0.27; SE=0.05; *P*<.001), passive media usage (*β*=0.31; SE=0.05; *P*<.001), and institutional media usage (*β*=0.16; SE=0.05; *P*<.001). These results indicate that higher levels of stress led participants to engage more frequently in various types of media usage. Of the 3 types of media usage, only active media usage was positively and significantly related to well-being (*β*=0.12; SE=0.04; *P*=.006). In contrast, passive media usage showed a negative association with well-being, approaching significance (*β*=–0.08; SE=0.05; *P*=.08). In conclusion, participants who engaged more in active media behaviors reported higher levels of well-being. Conversely, those who primarily engaged in passive media usage experienced a reduction in well-being.

**Figure 3:**
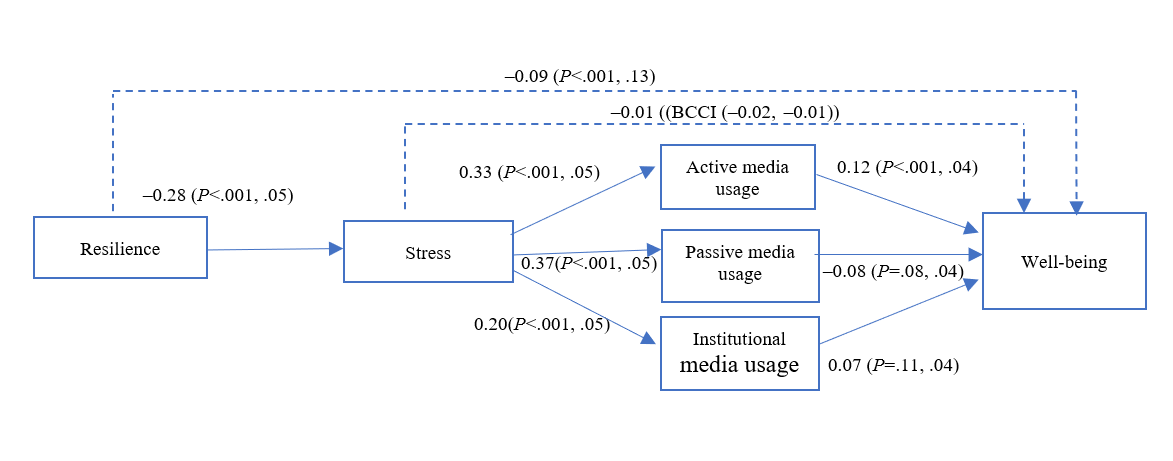
Results of the structural equation model. Unstandardized coefficients (B) are shown, with SEs in parentheses. BCCI: Bais corrected confidence interval.

The indirect relationship between resilience and well-being through stress and the 3 types of media usage was significant (indirect effect=0.09, 95% bias-corrected CI 0.05-0.13). However, when examining each path separately, we found that the source of the indirect connection was mediated specifically through active media behaviors (indirect effect=–0.01, 95% bias-corrected CI –0.02 to –0.01), while the other paths were not significant. These findings suggest that, in addition to the direct connection between resilience and well-being, resilience was negatively associated with stress. This stress reduction was associated with the use of active media behaviors, which positively impacted participants’ well-being. Figure 3 summarizes the model results.

Figure 3 depicts a path diagram of the SEM examining the relationships among resilience, stress, 3 types of media usage (active, passive, and institutional), and psychological well-being among Israeli adults (N=512) surveyed during the Israel–Hamas war (January 2024). Solid lines represent direct paths; dashed lines represent indirect (mediated) paths. Unstandardized coefficients (B) are reported for each path, with SEs in parentheses. Indirect effects are presented as standardized coefficients (*β*; Model fit: *χ*^2^_1_=1.5; *P*=.21; CFI=0.999; TLI=0.981; SRMR=0.013; RMSEA=0.033).

## Discussion

### Principal Findings

This study highlights the complex interplay between digital media engagement, psychological distress, and well-being during a national crisis. The findings show that high media engagement during wartime was associated with increased stress levels. This is consistent with the growing literature on “doomscrolling,” in which compulsive consumption of negative news has been associated with heightened anxiety [53]. Although seeking information is a natural response to uncertainty, the “vicious cycle” of constant monitoring may result in emotional exhaustion rather than relief [29]. Consistent with this broader pattern, higher exposure to COVID-19–related media information was associated with greater distress [18]. This pattern suggests that during times of crisis, media use may serve as a coping mechanism to manage uncertainty and maintain perceived control, a phenomenon also observed during the COVID-19 pandemic and in conflict-related contexts [18,19].

According to PMT, individuals adopt protective behaviors based on a threat- and coping-appraisal process [54]. However, our data suggest that during acute crises, heightened distress may be associated with increased digital engagement even when such engagement does not necessarily translate into psychological relief [15,18-20]. Viewed through PMT, stress can be interpreted as a proxy for heightened threat-related activation; our finding that stress was associated with greater engagement aligns with the general PMT expectation that perceived threat can increase motivation to act or seek coping responses. At the same time, the co-occurrence of elevated distress with both active and passive engagement patterns suggests that threat-related activation alone does not guarantee more adaptive outcomes, highlighting the potential importance of coping resources when interpreting crisis-related engagement. Accordingly, we interpret this pattern as increased engagement under distress, rather than as evidence of improved security behavior or decreased vigilance. Furthermore, prior research during population-level public health crises (eg, the COVID-19 pandemic) has examined links between anxiety and perceived efficacy of health-protective behaviors [16]. Related work has highlighted that exposure to the internet and media sources can be intertwined with health anxiety, health literacy, and information-seeking processes, including mediated pathways to downstream self-care behaviors [55]. Conceptual accounts suggest that media-induced uncertainty may sustain cycles of worry and repeated monitoring [29]. Empirical work using the Doomscrolling Scale indicates that doomscrolling is associated with psychological distress and poorer well-being, with distress mediating the relationship between doomscrolling and well-being indicators [56]. Continuous consumption of negative news, or doomscrolling, can be detrimental to mental health, especially when combined with intolerance of uncertainty and low psychological resilience [53].

The cluster analysis revealed 2 distinct engagement profiles: high and low media users. High engagement was associated with elevated stress levels and increased activity across digital platforms, particularly news consumption, social media, and online financial transactions. This pattern aligns with evidence from population-level crises showing that greater exposure to COVID-19–related media information is associated with higher psychological distress [18], and that social media use during the COVID-19 outbreak was associated with depression and secondary trauma [17]. In health-information contexts, exposure to the internet and media sources has also been linked to information-seeking and health anxiety processes [55]. This intensified reliance is consistent with the observation that the constant stream of updates can generate a sense of urgency and a feeling of permanent connectedness that has been linked to stress experiences [40]. Problematic smartphone-related experiences, such as nomophobia, have also been described among social media users [41]. Finally, qualitative work in individuals affected by social anxiety disorder suggests that unregulated digital engagement (eg, endless scrolling and information overload) may undermine subjective digital well-being [57].

Our findings further demonstrate that the type of media usage plays a critical role in associations with psychological outcomes. Active media use, characterized by interactive behaviors such as sharing posts or joining online groups, was positively associated with well-being, while passive media use (eg, browsing without interaction) showed a negative trend. These results align with previous studies suggesting that active media engagement may support coping and emotion regulation, whereas passive use may be associated with greater distress and emotional disengagement [58-60]. Specifically, active social media use has been linked to perceived online social support in some contexts [44]. Conversely, passive consumption has been associated with anxiety and negative self-image due to an increased likelihood of social comparison with peers [58,60]. More broadly, exposure to distressing or graphic crisis-related media content may amplify distress [7]. Institutional media usage (eg, accessing official or organizational channels) had a neutral to slightly positive effect, likely reflecting its role in fulfilling informational needs without causing emotional overload; however, the benefits of social networking services for information use can be attenuated by social media stress [61].

Path analysis confirmed that stress was significantly associated with all 3 types of media engagement. However, the indirect effects indicated that resilience was linked to well-being primarily through lower stress, while the specific indirect paths through media engagement types were small. These findings support the view that resilience is associated with better well-being and may buffer the effects of stress during crises, while engagement patterns may be differentially related to psychological outcomes [31,33,62]. Resilience is broadly defined as the fundamental human ability to “bounce back” or adapt successfully after exposure to a crisis or stressful situation [43]. Resilience has been implicated as a factor that may mitigate maladaptive engagement patterns, including doomscrolling, particularly under conditions of high anxiety and intolerance of uncertainty [53]. Moreover, strengthening resilience at individual and societal levels is recognized as an important strategy for coping with large-scale crises and disasters [31,63].

Importantly, cyber-related knowledge did not significantly differentiate between the 2 engagement clusters. This suggests that during acute crises, emotional states (particularly stress) may play a more prominent role than technical background variables in shaping digital media engagement patterns. Consistent with scholarship on anxiety and decision-making, intense anxiety or panic may impair deliberative decision-making and coherent preference ordering [30], and high-arousal negative emotions (eg, fear and anger) can bias risk judgments and subsequent choices [64]. A critical implication of this emotional overload is the onset of “cybersecurity fatigue,” a state of mental and emotional exhaustion that contributes to burnout and increased psychological strain, potentially leading users to consciously or unconsciously neglect essential security protocols [65,66]. Similar findings have been reported in the cybersecurity behavior literature, where perceived vulnerability and emotional responses influenced behavior more strongly than technical knowledge alone [67,68]. This underscores the importance of integrating emotional and psychological components into digital literacy programs, especially for populations at risk of high exposure to crisis media.

From a theoretical perspective, these findings can be interpreted using PMT as a conceptual lens. While PMT traditionally focuses on cognitive threat and coping appraisals [69], our results highlight associations between stress and digital media engagement, showing that higher stress was associated with greater engagement across types, whereas engagement patterns showed different associations with well-being. Accordingly, we avoid concluding that stress motivates protective or maladaptive behaviors and instead describe these as observed associations that may vary by context. Prior PMT-informed cybersecurity research illustrates that protective behavior is typically framed as something that can be shaped through modifiable organizational levers, such as clear policies and security education, training, and awareness programs, which are intended to strengthen coping appraisals and translate perceived threat into protective action [70]. PMT reviews further emphasize that threat appraisal alone is insufficient; protective responding is expected when coping appraisal, such as perceived efficacy and self-efficacy, is strong relative to perceived costs [69]. Interpreted through this lens, our finding that higher stress co-occurred with both active and passive engagement patterns suggests that crisis-related arousal may broadly increase engagement, whereas coping resources, such as resilience, may be particularly important in determining whether engagement is linked to more favorable psychological outcomes. More broadly, crisis-specific frameworks emphasize the role of information and communication technology–mediated information environments in crisis management and communication [71]. In this study, resilience was associated with lower stress and higher well-being, suggesting that coping resources are relevant when interpreting crisis-related engagement and outcomes [43]. In the context of doomscrolling, intolerance of uncertainty and resilience-related processes have been discussed as relevant correlates [53]. Overall, these findings support a cautious, theory-informed interpretation without implying causal mechanisms or model refinement beyond the variables measured in this study.

The findings have practical implications for designing public health and digital communication strategies during emergencies. Campaigns should encourage intentional and participatory media use, such as verifying sources, seeking community connection, and sharing factual information, while raising awareness about the mental health risks of excessive passive consumption. Platforms could be adapted to promote active engagement by prompting users toward verified resources or facilitating moderated peer-support communities. Educational and organizational programs that combine emotion regulation, media literacy, and resilience-building may serve as protective interventions, particularly for populations frequently exposed to distressing crisis-related media content. Evidence syntheses suggest that media literacy programs can strengthen critical evaluation of information and reduce susceptibility to misinformation [72]. A recent meta-analysis found that media literacy interventions improved fake news credibility assessment, suggesting that strengthening credibility judgment skills through structured media literacy training may support safer digital decision-making during crises, including decisions about sharing, trusting, or acting on rapidly circulating claims [73]. The development of digital tools, including mobile apps to support mental health response in disasters and remotely delivered mental health services, may strengthen mental health support and continuity of care when in-person services are disrupted by extending access to support across preparedness, response, and recovery phases, particularly when demand is high and resources are constrained [74,75].

### Strengths and Limitations

This study presents several important strengths along with acknowledged limitations. A key strength is its focus on the understudied intersection of cybersecurity-related behavior, media engagement, and psychological well-being during an active national crisis. Moreover, the contribution of this study lies in its unique context, which enabled the observation of digital behaviors during an acute and ongoing national crisis. While previous research has explored media consumption during public health crises such as the COVID-19 pandemic [58] and has documented the psychological toll of exposure to graphic war-related content [7], this study extends this literature in several important ways.

First, rather than treating media usage as a unidimensional construct, we empirically identified 3 distinct patterns of digital engagement: active, passive, and institutional usage, each with different implications for psychological well-being. Second, by embedding these patterns within a stress-resilience framework, we demonstrated that the relationship between media engagement and well-being is not uniform but depends critically on the type of engagement. Specifically, active participation in digital platforms was associated with better well-being, consistent with the view that such behaviors may serve as a form of emotion-focused coping and social support-seeking during acute crisis [76]. In contrast, passive consumption showed a negative trend, aligning with prior findings on doomscrolling and emotional exhaustion [53]. These findings highlight the importance of moving beyond aggregate measures of screen time or media exposure and instead attending to the qualitative nature of digital engagement during wartime.

A further contribution of this study lies in its broader conceptualization of digital media usage. Unlike previous studies, which have typically focused on social media platforms (eg, Abbas et al [77]; Huckins et al [78]; Gao et al [79]), this study also examined institutional digital behaviors, including online banking and access to government and public authority websites. In an era of accelerating digitalization, digital applications are no longer limited to entertainment and social interaction, they have increasingly become the primary interface between individuals and institutional agents, such as financial institutions and governmental services. This shift may have important implications for cybersecurity, as greater reliance on digital institutional channels during crisis periods may heighten individuals’ vulnerability to cyberattacks. Enhanced cybersecurity awareness and digital literacy are therefore particularly critical during times of crisis, such as war or a pandemic, when the availability of in-person institutional contact is restricted and individuals are compelled to rely more heavily on digital alternatives. In this study, we therefore expand the scope of inquiry beyond social media, examining whether individuals turn to digital applications not only to seek information and social connection but also to manage practical and institutional needs during wartime.

While the findings provide important insights, several limitations should be considered when interpreting the results. First, the cross-sectional design precludes causal inference [80]. Although the path analysis model implies directionality, the temporal order between stress, media engagement, and well-being cannot be established from a single time-point measurement. All associations reported in this study should therefore be interpreted as correlational rather than causal. Longitudinal or experimental designs are needed to establish whether stress causally drives media engagement or whether intensive media use itself amplifies stress over time.

Second, cybersecurity awareness and knowledge were assessed using a single self-report item. In addition, media usage was operationalized directly and did not rely on previously validated instruments. While this approach captures behavior directly, this operationalization may not fully reflect the multidimensional nature of media usage awareness, which may also involve more implicit or less visible patterns of engagement. Future studies should incorporate validated measures to enable more rigorous testing of their role in shaping digital behavior during crises.

Third, the study did not measure potential confounding variables that may influence both media engagement and well-being, such as prior mental health history, social support networks, proximity to conflict zones, or direct exposure to wartime events. The observed associations between stress, media use, and well-being may therefore partly reflect the influence of unmeasured third variables rather than the relationships among the constructs themselves.

Lastly, participants were recruited through an online panel using quota sampling, which, while designed to approximate the demographic distribution of the Israeli population, does not constitute a probability sample. Self-selection bias [81] may have resulted in the overrepresentation of individuals who are more digitally engaged or more willing to report their media behaviors and psychological states. In addition, previous studies suggest that cybersecurity behaviors differ across cultures [82,83]. Accordingly, the findings should be interpreted with caution and may not be fully generalizable to populations with lower digital access or different media consumption patterns. In this vein, it should also be noted that the study was conducted within a single national context during the early months of the Israel–Hamas war, which limits the external validity of the findings. The specific cultural, political, and media environment of this conflict may not be representative of other crisis contexts, such as natural disasters, pandemics, or conflicts in different geopolitical settings. Replication across diverse crisis contexts and cultural settings is therefore required before broader conclusions regarding the relationship between stress, media engagement, and well-being can be drawn. Future research should build on these findings by examining the dynamic relationships among stress, media engagement, and well-being using longitudinal designs and more comprehensive measures of digital behavior.

### Future Directions

Future research should test the direction and mechanisms underlying the observed associations by using longitudinal and experience-sampling designs that capture within-person changes in stress, resilience, and engagement over time and across crisis phases. Studies that combine self-reports with objective indicators of digital behavior (such as passive sensing or platform-provided usage metrics) and more detailed characterization of content (such as exposure to graphic material, misinformation, or institutional alerts) may help distinguish when engagement is adaptive vs burdensome. It is also important to examine heterogeneity across subgroups (ie, age, prior trauma, frontline exposure, and baseline mental health) and across platforms and engagement modes, including institutional channels that are often overlooked. Finally, intervention-focused research should evaluate targeted, real-world strategies (ie, media literacy and resilience-building components embedded in crisis communication, platform-based prompts toward verified sources, and moderated peer-support pathways) to determine which approaches most effectively promote healthier engagement and protect well-being during prolonged crises.

### Conclusions

This study offers new evidence on wartime digital media engagement by moving beyond time-based exposure measures and empirically identifying distinct engagement patterns, including active, passive, and institutional modes, which are rarely examined together in crisis-media research. Compared with previous work that often focuses on overall media exposure or social media alone, our findings offer a more detailed account of how different forms of engagement relate to stress, resilience, and well-being during an ongoing armed conflict. This contribution advances the field by presenting an empirically grounded framework for interpreting when engagement may be supportive vs potentially burdensome in prolonged crises. Practically, the results can inform public health and organizational communication by emphasizing targeted guidance that encourages adaptive, intentional engagement (ie, seeking verified information and social support) while reducing risks from excessive passive exposure. They can also help platforms and service providers direct users to reliable institutional information and supportive resources.

## Data Availability

The data sets generated and analyzed during this study are available from the corresponding author upon reasonable request, subject to approval by the Ethics Committee of Ariel University.

## Abbreviations

CFI: comparative fit index
TLI: Tucker-Lewis Index
SRMR: standardized root-mean-square residual
EFA: exploratory factor analysis
GAD-7: Generalized Anxiety Disorder-7 Scale
KMO: Kaiser-Meyer-Olkin
PHQ-9: Patient Health Questionnaire-9
PMT: protection motivation theory
PTSD: posttraumatic stress disorder
RMSEA: root-mean-square error of approximation
RQ: research question
SEM: structural equation modeling
